# The Relationship Between CCN3 and Rheumatoid Arthritis and Its Potential Therapeutic Value

**DOI:** 10.1155/jimr/8846865

**Published:** 2026-07-05

**Authors:** Ye Yuan, Hui Yin, Mengya Jiao, Lihua Duan

**Affiliations:** ^1^ Department of Microbiology and Immunology, School of Basic Medicine, Guangdong Pharmaceutical University, Guangzhou, 510006, China, gdpu.edu.cn; ^2^ Jiangxi Province Key Laboratory of Immunology and Inflammation, Jiangxi Provincial People’s Hospital, Nanchang, 330006, China, jxsrmyy.cn; ^3^ JXHC Key Laboratory of Rheumatology and Immunology, Jiangxi Provincial People’s Hospital, Nanchang, 330006, China, jxsrmyy.cn; ^4^ Department of Rheumatology and Clinical Immunology, Jiangxi Provincial People’s Hospital, The First Affiliated Hospital of Nanchang Medical College, Nanchang, 330006, China, jxsrmyy.cn

**Keywords:** CCN3, immune regulation, inflammation, joint destruction, rheumatoid arthritis

## Abstract

Rheumatoid arthritis (RA) is a chronic autoimmune disease characterized by persistent synovial inflammation, progressive joint destruction, and bone damage. Despite advances in disease‐modifying antirheumatic drugs (DMARDs), unmet clinical needs arising from incomplete efficacy and safety concerns continue to drive the search for innovative therapeutic targets. Cellular communication network factor 3 (CCN3), a matricellular CCN family protein, is low in normal synovium but elevated in RA synovium and circulation, where it correlates with disease activity, anti‐cyclic citrullinated peptide (anti‐CCP) antibody titers, and IL‐6. CCN3‐associated effects may involve inflammatory‐cell recruitment, fibroblast‐like synoviocyte (FLS) activation and senescence, extracellular matrix (ECM) remodeling, cartilage degeneration, and osteoclastogenesis. Mechanistically, CCN3 may interact with RA‐relevant NF‐κB, Wnt/β‐catenin, BMP/Smad, PI3K/Akt/mTOR, IL‐6/JAK/STAT, Notch, and MAPK signaling networks. This review comprehensively examines the expression patterns, functional mechanisms, signaling pathways, and ongoing debates concerning CCN3 in RA and further explores its promise as a therapeutic target in preclinical research.

## 1. Introduction

Rheumatoid arthritis (RA) is a systemic autoimmune disease characterized by chronic synovitis, pannus formation, lymphocytic infiltration, and progressive cartilage and bone destruction, and it may also involve the heart, lungs, eyes, skin, and peripheral nerves [1]. RA follows a relapsing‐remitting course and, if left untreated, may cause irreversible joint damage and systemic complications, including hematologic abnormalities, interstitial lung disease, and pleural effusion [2, 3]. Its pathogenesis is driven mainly by immune dysregulation and activation of inflammatory cytokine networks [4, 5]. Infectious triggers may activate antigen‐presenting cells and autoreactive lymphocytes, thereby breaking immune tolerance and promoting the release of proinflammatory cytokines such as TNF‐α and IL‐6, which amplify synovial inflammation and contribute to tissue and organ damage [6].

At present, disease‐modifying antirheumatic drugs (DMARDs) remain the cornerstone of clinical management in RA. Although a subset of patients respond favorably to methotrexate monotherapy, the majority require combination therapy involving methotrexate and targeted agents [7]. However, current targeted therapies continue to face the dual challenges of limited efficacy and safety concerns. With growing insights into the immunopathogenesis of RA, treatment strategies have progressively shifted from mere suppression of inflammation to precision interventions tailored to individual pathogenic pathways. This paradigm shift underscores the importance of identifying novel therapeutic targets and biomarkers to optimize clinical decision‐making and facilitate patient stratification. Cellular communication network factor 3 (CCN3), a member of the CCN protein family, exhibits a unique subcellular distribution across cytoplasmic, nuclear, and extracellular compartments. Through matricellular signaling networks, it exerts diverse regulatory roles in embryonic development, fibrosis, inflammatory responses, and tumorigenesis [8–10]. Recent studies have indicated that CCN3 possesses critical biological functions in various immune‐related diseases [11], particularly suggesting a potential involvement in the inflammatory processes and joint destruction characteristic of RA. This review aims to systematically elucidate the mechanistic role of CCN3 in RA pathogenesis and assess its translational value as a novel therapeutic target.

## 2. Methods

**Figure 1:**
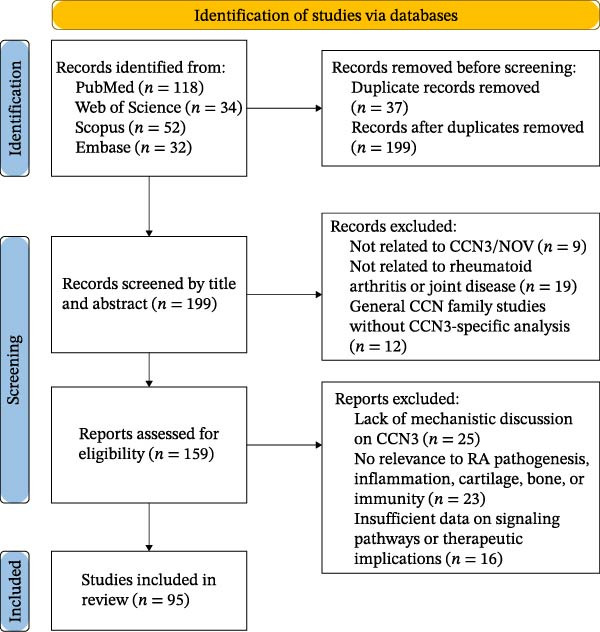
PRISMA flow diagram.

This review summarizes the potential role of CCN3 in RA pathogenesis and evaluates its translational relevance as a therapeutic target. A systematic search was performed in PubMed, Web of Science, Scopus, and Embase for articles published from 1990 to the present. The search terms included: “CCN3,” “NOV,” “cellular communication network factor 3,” “rheumatoid arthritis,” “synoviocytes,” “osteogenesis,” and “signaling pathways.” Inclusion criteria were [1] original articles or reviews published in English [2]; studies investigating the role of CCN3 in RA, synoviocytes, or bone metabolism. Exclusion criteria were [1] studies not related to CCN3, RA, or joint diseaseand [2] studies lacking CCN3‐specific analysis, mechanistic discussion, or sufficient signaling pathway data. After duplicate removal, records were screened by title, abstract, and full text based on the above criteria. The selection process is summarized in the PRISMA flow diagram (Figure 1).

## 3. CCN Family and CCN3

### 3.1. CCN Family

The CCN family comprises six secreted matricellular proteins, designated CCN1‐CCN6, that regulate diverse cellular and tissue responses under physiological and pathological conditions [12, 13]. CCN1−3 were first identified as CYR61, CTGF, and NOV, whereas CCN4−6 correspond to WISP‐1, WISP‐2, and WISP‐3. Most CCN proteins contain four conserved domains: an insulin‐like growth factor‐binding protein domain, a von Willebrand factor type C domain, a thrombospondin type 1 repeat, and a cysteine knot‐containing C‐terminal domain, with CCN5 lacking the C‐terminal domain [11, 14]. Functionally, CCN proteins regulate angiogenesis, tissue repair, inflammation, and extracellular matrix (ECM) remodeling by modulating cell proliferation, adhesion, migration, and differentiation [14].

### 3.2. Structure and Biological Functions of CCN3

CCN3 was initially identified in 1992 as NOV, a viral integration gene in avian nephroblastoma [15]. Subsequent studies have demonstrated its broad expression in mammalian endothelial cells, smooth muscle cells, fibroblasts, chondrocytes, and regulatory T (Treg) cells, implicating its involvement in vascular homeostasis, immune modulation, and ECM remodeling [16–18]. In contrast to CCN1 and CCN2, although CCN3‐knockout mice are viable, they exhibit tissue‐specific defects such as skeletal abnormalities, indicating that CCN3 plays an indispensable regulatory role in postnatal tissue homeostasis and pathological processes [11]. As a structurally conserved yet functionally versatile member of the CCN family, CCN3 possesses a four‐domain modular architecture that endows it with a unique “molecular switch” capability, allowing the dynamic modulation of cell behavior in diverse microenvironments.

CCN3 exerts cell type‐specific effects. It promotes endothelial adhesion and migration through integrins αvβ3, α6β1, and α5β1 but suppresses proliferation and migration in smooth muscle cells and shows antiproliferative activity in some tumor cells [11, 16, 17]. CCN3 is also regulated by inflammatory cytokines, including TNF‐α and IL‐1β, and can in turn modulate inflammatory mediator production and matrix‐degrading enzyme expression [12, 19, 20]. In astrocytes, CCN3 regulates cytokine expression through integrin β1/Rho/ROCK/c‐Jun N‐terminal kinase (JNK)/NF‐κB and integrin β5/Rho/ROCK/p38/NF‐κB signaling pathways [21]. By regulating osteoblast and chondrocyte differentiation, CCN3 further contributes to skeletal and cartilage homeostasis. Collectively, these findings support CCN3 as a context‐dependent matricellular regulator of inflammation, immune responses, and ECM remodeling.

## 4. CCN3 and RA

### 4.1. Overview of the Pathogenesis of RA

**Figure 2:**
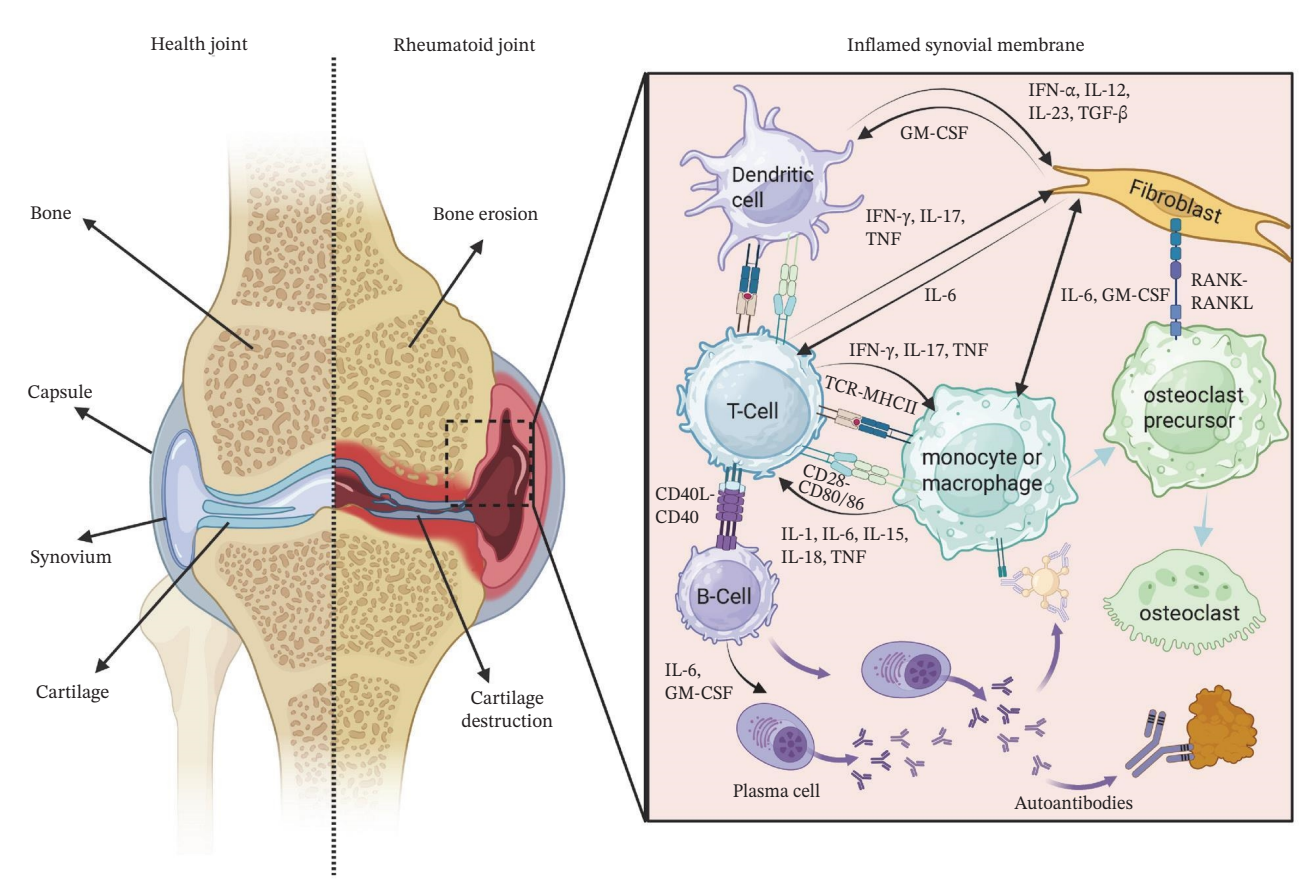
Comparison between normal and rheumatoid joints. In RA, the innate and adaptive immune systems are dysregulated under the combined influence of genetic and environmental factors. Synovial‐resident immune and stromal cells, along with their secreted cytokines, play central roles in RA pathogenesis. Dendritic cells, macrophages, and B cells contribute to disease initiation by presenting modified self‐antigens to T cells via class II major histocompatibility complex (MHC) molecules and costimulatory signals, while producing cytokines such as interleukin (IL)‐12, IL‐15, and IL‐18 that promote T‐cell activation and differentiation. The resulting effector T cells release cytokines that further activate monocytes, macrophages, and fibroblasts, creating a self‐amplifying inflammatory loop. B cells differentiate into antibody‐producing plasma cells and contribute to autoantibody production. Synovial fibroblasts express receptor activator of nuclear factor κB ligand (RANKL), which induces monocyte‐to‐osteoclast differentiation, ultimately leading to bone resorption. GM‐CSF, granulocyte–macrophage colony‐stimulating factor; IFN, interferon; IL, interleukin; MHC, major histocompatibility complex; TCR, T‐cell receptor; TGF, transforming growth factor; TNF, tumor necrosis factor.

RA is a complex autoimmune disease characterized primarily by symmetric polyarthritis, with a global prevalence estimated at ~0.5%–1% [22]. A higher geographic incidence has been reported in regions such as North America, Japan, and Argentina [22, 23]. The disease predominantly affects individuals over the age of 40, with a significantly higher prevalence observed in women compared to that in men [24]. Disease progression is characterized by dysregulation of both innate and adaptive immune responses, culminating in persistent synovial inflammation and eventual joint destruction [25–27]. Activated immune cells such as CD4^+^ and CD8^+^ T cells, B cells, plasma cells, macrophages [28], and dendritic cells interact with synovial fibroblasts to form a complex inflammatory network (Figure 2). Through the release of proinflammatory cytokines such as TNF‐α, IL‐6, and IL‐17, along with matrix‐degrading enzymes, this network promotes synovial hyperplasia, pannus formation, and progressive destruction of cartilage and bone [29]. At the molecular level, multiple signaling pathways, including NF‐κB, MAPK, JAK/STAT, Wnt/β‐catenin, and PI3K/Akt, are intricately interconnected to regulate key pathological processes such as inflammation, cell proliferation and apoptosis, and tissue remodeling. These pathways not only sustain chronic inflammatory responses but also amplify tissue injury through self‐reinforcing feedback loops. Despite ongoing advances in our understanding of RA pathogenesis, there remains a lack of reliable biomarkers capable of accurately predicting disease onset, assessing activity, or forecasting therapeutic response. Accordingly, elucidating the initiation and perpetuation mechanisms of RA remains a critical scientific priority in the field of rheumatology.

### 4.2. Expression Pattern of CCN3 in RA

#### 4.2.1. CCN3 Expression in Tissues From Patients With RA

Komatsu et al. [30] reported, via in situ hybridization, that CCN3 expression is nearly undetectable in normal synovial and cartilage tissues but is markedly upregulated in the synovium of patients with RA and osteoarthritis (OA). Specifically, weak CCN3 signals were detected in the superficial and stromal layers of synovial tissue from RA knee joints and OA hip joints, whereas no expression was detected in healthy synovium. Likewise, no appreciable CCN3 signal was observed in the cartilage of RA or OA patients. These findings suggest that CCN3 is primarily produced by the hyperplastic synovial tissue rather than by articular cartilage. This observation was further supported by immunohistochemical analysis, which showed markedly increased CCN3 protein accumulation in the RA synovial tissue compared to OA controls [31]. The presence of CCN3 in the inflamed RA synovium represents a crucial starting point for elucidating its functional role in RA pathogenesis.

#### 4.2.2. Circulating Levels of CCN3 in Patients With RA

Wei et al. [31] measured serum CCN3 levels in patients with RA using enzyme‐linked immunosorbent assay (ELISA) and observed a significant increase compared to healthy controls. Notably, circulating CCN3 levels showed a positive correlation with disease activity, as indicated by a significant association with the disease activity score in 28 joints (DAS28), calculated using either erythrocyte sedimentation rate (ESR) or C‐reactive protein (CRP). Patients with elevated serum CCN3 levels tended to exhibit a higher disease activity. Additionally, CCN3 levels were positively associated with titers of anti‐cyclic citrullinated peptide (anti‐CCP) antibodies, a highly specific serological marker for RA. Interestingly, no significant correlation was found between CCN3 levels and rheumatoid factor (RF) titers. In contrast, a strong positive correlation was observed with serum IL‐6 levels, suggesting that CCN3 may reflect both the inflammatory burden and autoimmune activity in RA. Collectively, current evidence consistently demonstrates that CCN3 expression is upregulated in RA, both locally in the synovial tissue and systemically in the circulation [32, 33]. CCN3 levels correlate with disease severity, suggesting a potential pathogenic role and supporting its utility as a candidate biomarker for monitoring disease activity in RA.

### 4.3. Functional Roles of CCN3 in RA

**Figure 3:**
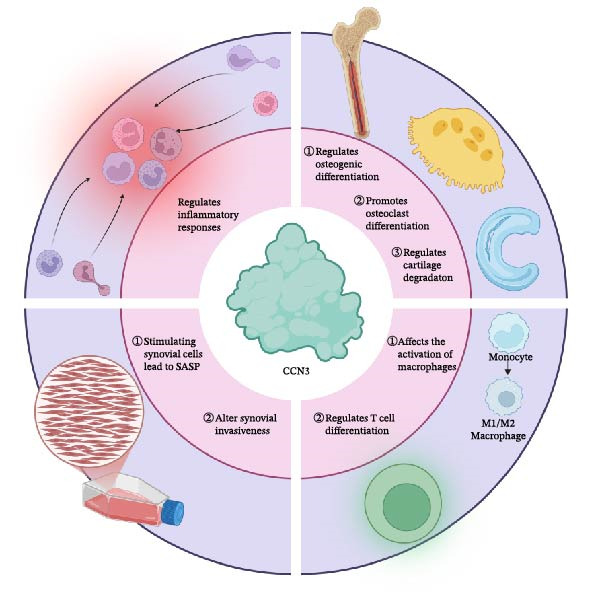
Pathogenic roles of CCN3 in RA. CCN3 contributes to the pathogenesis of RA through multiple mechanisms. Proinflammatory effects: CCN3 promotes migration and adhesion of inflammatory cells and angiogenesis, and amplifies the production of proinflammatory cytokines (e.g., IL‐6 and IL‐1β) via activation of the NF‐κB signaling pathway. Synovial pathology: CCN3 may enhance the invasiveness of FLS and activate FLS senescence pathways and the senescence‐associated secretory phenotype (SASP), thereby exacerbating inflammation. Bone destruction: although CCN3 has been shown to exert cartilage‐protective effects in osteoarthritis, evidence in RA primarily supports its role in promoting osteoclast differentiation and bone erosion. Immunomodulation: as a marker of chronic inflammation, CCN3 may regulate immune responses indirectly by influencing macrophage polarization and modulating the Treg/Th17 balance.

CCN3 participates in multiple pathological processes involved in RA, including inflammatory responses, synovial pathology, articular cartilage destruction, and immune regulation (Figure 3).

#### 4.3.1. Regulation of Inflammatory Responses

The transendothelial migration of inflammatory cells is a key multistep process in the initiation and propagation of inflammation. CCN family members, as matricellular proteins, promote the adhesion and migration of inflammation‐related cells, such as monocytes and macrophages, through integrin‐dependent mechanisms [34, 35]. Additionally, CCN3 induces angiogenesis in vivo by promoting endothelial cell proliferation and survival [16], thereby contributing to persistent and progressive inflammation. Notably, CCN proteins can serve as substrates for activated endothelial cells to mediate platelet adhesion, an essential feature of their function in inflammatory microenvironments. Cytokines and other inflammatory mediators play central roles throughout all phases of the inflammatory response [36], while highly expressed CCN3 in RA joints indicates its potential involvement in regulating local inflammation. Current evidence supports a bidirectional interaction between CCN3 and classical inflammatory mediators. Serum CCN3 levels in RA patients are positively correlated with proinflammatory cytokines such as IL‐6 [31], suggesting a link between elevated CCN3 expression and increased inflammatory activity. In vitro studies further show that silencing CCN3 in a lipopolysaccharide (LPS)‐induced alveolar epithelial inflammation model significantly reduces IL‐1β and TGF‐β1 levels [37], supporting its role in sustaining or amplifying inflammatory mediator production, which is consistent with the report by Chen et al. [38] Mechanistically, CCN3 promotes nuclear translocation of the NF‐κB p65 subunit, thereby enhancing the transcription of downstream proinflammatory genes. Since the NF‐κB pathway is a central regulator of cytokines such as TNF‐α and IL‐1β in the RA synovium [39], these findings suggest that CCN3 may contribute to persistent synovial inflammation by enhancing NF‐κB signaling. Although some studies suggest that CCN3 may exert anti‐inflammatory effects under certain conditions, such as its proposed negative regulation of specific inflammatory factors in RA reported by Lin et al. [40], direct supporting evidence remains limited. Overall, most current studies support a proinflammatory role for CCN3 in RA. Its expression is induced by inflammatory stimuli and, through positive feedback, promotes the production of key cytokines, such as IL‐6 and IL‐1β. Moreover, CCN3 may engage multiple signaling pathways, including MAPK and NF‐κB, to amplify the inflammatory response and contribute to the chronic inflammation characteristic of RA.

#### 4.3.2. Effects of CCN3 on Synovial Cells

The synovial tissue is primarily composed of fibroblast‐like synoviocytes (FLSs) and their associated ECM. In healthy joints, FLS form the synovial lining and play a crucial role in maintaining joint homeostasis by secreting ECM components, including hyaluronic acid, lubricin, and various collagens [41–43]. In RA, citrullinated ECM components can trigger the production of anti‐citrullinated protein antibodies (ACPAs), which in turn drive the transformation of normal FLS into an aggressive RA‐FLS phenotype. These pathogenic RA‐FLS display hyperproliferative and invasive properties, contributing to synovial hyperplasia and joint destruction [41, 44]. Emerging evidence indicates that CCN3 may regulate FLS adhesion and migration through integrin–heparan sulfate proteoglycan (HSPG) interaction [14, 45, 46]. Owing to its conserved structural domains and shared functions within the CCN family, CCN3 is hypothesized to modulate the invasive RA‐FLS phenotype, although further validation using gene‐targeted approaches in primary FLS models is required. Beyond its effects on migration and invasion, CCN3 also activates cellular senescence pathways in synovial cells [47]. In vitro studies show that recombinant CCN3 stimulation significantly upregulates TGF‐β signaling and p53‐associated genes in FLS, including ADGRB1 and PCBP4. The latter, along with ZNF385A, is implicated in the regulation of cellular senescence. Venn diagram analysis revealed that 20 CCN3‐upregulated genes overlapped with established senescence‐associated gene signatures, accompanied by increased expression of markers typical of the senescence‐associated secretory phenotype (SASP). In vivo studies further confirmed that intra‐articular CCN3 injection significantly increased the proportion of p53‐positive cells in the murine knee synovium. These findings suggest that CCN3 contributes to a maladaptive cycle of chronic inflammation and immunosenescence in RA by inducing FLS senescence and SASP activation, thereby accelerating disease progression [48].

#### 4.3.3. The Role of CCN3 in Cartilage Degradation and Joint Destruction

Cartilage degradation and bone erosion are key pathological features of RA; however, direct evidence linking CCN3 to RA‐related cartilage and bone damage remains limited. Evidence from OA studies suggests that CCN3 contributes to cartilage homeostasis, as CCN3 is upregulated in OA cartilage and CCN3‐deficient mice spontaneously develop severe OA‐like joint degeneration [49–51]. In the MIA‐induced cartilage injury model, intra‐articular injection of recombinant CCN3 alleviated cartilage degradation, partly by inhibiting IL‐1β‐induced PI3K/Akt/mTOR activation, HMGB1 expression, and matrix metalloproteinases (MMPs) production while promoting proteoglycan and lubricin synthesis [52, 53]. In bone metabolism, CCN3 acts mainly as a negative regulator of osteogenesis by interfering with BMP and Notch signaling, supported by increased Smad1/5 phosphorylation in CCN3‐deficient mice [54]. Conversely, excessive CCN3 may promote cartilage aging, as H_2_O_2_‐induced senescence increases CCN3 expression through the p53/p21 axis, and cartilage‐specific CCN3 overexpression causes early knee joint degeneration [55]. CCN3 also promotes tenascin‐C expression and suppresses type‐X collagen synthesis, indicating a role in maintaining the articular chondrocyte phenotype [56]. Together, these findings suggest that CCN3 maintains cartilage integrity under physiological conditions but may contribute to cartilage senescence and degeneration when dysregulated.

A hallmark histopathological feature of RA is the proliferation of invasive pannus tissue at the interface of the synovium, cartilage, and bone. This hyperplastic tissue, known as the pannus, extends over the articular cartilage surface, impairing the synovium’s protective function [57, 58] and facilitating adhesion and invasion by FLS. FLS secrete MMPs, which degrade the collagen network and diminish the cartilage’s ability to retain glycosaminoglycans and water, thereby compromising its structural integrity. In addition to cartilage degradation, bone erosion represents a key pathological process in RA. Proliferating synovial fibroblasts, along with activated T and B lymphocyte subsets, contribute to bone resorption by expressing the receptor activator of nuclear factor κB ligand (RANKL). Together with proinflammatory cytokines, particularly TNF and IL‐6, RANKL promotes osteoclast differentiation. Mature osteoclasts adhere to bone surfaces, create an acidic microenvironment, and initiate bone matrix degradation [22]. Previous studies have shown a strong association between CCN3 expression and key proinflammatory cytokines in RA, suggesting that CCN3 may facilitate osteoclastogenesis by modulating the crosstalk between inflammatory cytokines and RANKL signaling [39]. Supporting this hypothesis, Tokuhiro et al. [47] reported that stimulating human FLS with recombinant CCN3 led to increased expression of key osteoclastogenic transcription factors, including NFATc1 and AP‐1. Furthermore, stimulation of human monocytes with CCN3, followed by TRAP staining and actin ring analysis, confirmed that CCN3 promotes osteoclast formation. In additional experiments, human monocytes cultured on dentin slices and treated with CCN3 displayed gene expression profiles indicative of osteoclast differentiation. Collectively, these findings suggest that CCN3 contributes to RA‐associated joint damage by both indirectly facilitating bone destruction through inflammatory signaling and directly promoting osteoclast differentiation and function.

#### 4.3.4. Immunoregulatory Role of CCN3 in RA

Dysregulated immune cell activity, including that of T cells, B cells, and monocytes/macrophages, is a major contributor to joint destruction in the pathogenesis of RA. As a molecule implicated in various immune‐related diseases, CCN3 may exert both direct and indirect effects on immune cell functions. Notably, CCN3 expression is frequently upregulated in chronic inflammatory conditions, including atherosclerosis, RA, and chronic liver disease [11, 59–61], suggesting its role in immune cell‐mediated chronic inflammation. Specifically, studies have shown that CCN3 regulates macrophage polarization toward both M1 and M2 phenotypes [61], contributing to lipid uptake and foam cell formation in atherosclerosis. Consistently, CCN3 has been reported to promote M2 macrophage infiltration in prostate cancer and enhance vascular endothelial growth factor (VEGF) expression through activation of the FAK/Akt/NF‐κB pathway [62], indicating its capacity to modulate macrophage behavior. Given that macrophages are the predominant proinflammatory effector cells in the RA synovium, CCN3‐mediated enhancement of NF‐κB signaling may amplify their cytokine‐producing capacity. Although direct evidence is lacking, indirect observations suggest that CCN3 may play a regulatory role in T‐cell differentiation and function. In the central nervous system (CNS), CCN3 has been identified as a novel Treg‐derived factor that promotes oligodendrocyte differentiation and myelination [18], with functional implications in disorders such as multiple sclerosis [63]. IL‐6, secreted by synovial fibroblasts and B cells in RA [64, 65], plays a pivotal role in regulating the differentiation of Tregs, T helper 17 (Th17) cells, and follicular helper T (Tfh) cells. The IL‐6–STAT3 signaling axis is essential for Th17 development, and its hyperactivation promotes IL‐17 A‐dependent autoimmune arthritis in murine models [39]. The critical role of STAT3 in human Th17 differentiation has also been validated in clinical studies [66]. At the molecular level, IL‐6 activates STAT3, which suppresses Foxp3 expression and reprograms Tregs toward a Th17‐like phenotype through epigenetic remodeling [67, 68]. Given the positive correlation between CCN3 and IL‐6 levels in RA patients [31], CCN3 may plausibly influence the Treg/Th17 balance indirectly, thereby contributing to immune dysregulation in RA. Collectively, these findings suggest that CCN3 modulates immune cell function by shaping the inflammatory microenvironment. Although not a classical cytokine or chemokine, CCN3, a matricellular protein, can modulate local cell–matrix interactions and intracellular signaling pathways, thereby indirectly regulating both the magnitude and quality of the immune response in RA.

### 4.4. CCN3‐Associated Signaling Pathways in RA

The pathogenesis of RA involves the aberrant activation of multiple intracellular signaling pathways. As an extracellular regulatory molecule, CCN3 may engage with or modulate these pathways, thereby influencing inflammatory responses and tissue remodeling (Table 1).

**Table 1:** Functional roles of CCN3 in RA‐associated signaling pathways.

Signal path	Reference	Function of CCN3
NF‐κB	[16, 37]	Regulation of IκBα degradation and p65 nuclear translocation.

Wnt/β‐catenin	[69]	Indirect inhibition of β‐catenin levels and transactivation in osteoblasts via binding to BMP‐2.
	[70]	Direct suppression of pathway activity via downregulation of LEF1/TCF.

PI3K/Akt	[52, 71]	Modulation of PI3K, AKT, and mTOR phosphorylation levels.

JAK/STAT	[72]	Activation of JAK family kinases induces nuclear translocation of STAT3 homodimers and subsequent transcriptional activation of downstream target genes (e.g., RANKL, MMP‐9).

TGF‐β	[47, 73, 74]	Facilitation of TGF‐β signaling and cyclin‐dependent kinase inhibitor interactions mediates senescence pathway activation in macrophages and synoviocytes.
	[75]	Promotion of TGF‐β signaling contributes to activation of the noncanonical osteoclast differentiation pathway, resulting in bone damage.

Notch	[54, 69, 76–78]	By modulating signaling pathway function through the extracellular domain of Notch1 in osteoblasts. By inhibiting Notch signaling through DLL1 suppression.

MAPK	[79]	By reducing phosphorylation of ERK1/2 and p38 MAPK in osteoblasts to suppress this pathway.

#### 4.4.1. NF‐κB Signaling Pathway

In RA, aberrant NF‐κB activation drives disease progression by promoting proinflammatory cytokine production, innate immune activation, immune cell recruitment, and T‐ and B‐cell responses [80–82]. CCN3 has been shown to regulate NF‐κB signaling in several non‐RA models, including prostate cancer, hepatocellular carcinoma, and alveolar epithelial cells [37, 60, 83]. In alveolar epithelial cells, inflammatory stimulation induces NF‐κB‐dependent CCN3 expression, whereas CCN3 further promotes p65 nuclear translocation, suggesting a positive feedback loop between CCN3 and NF‐κB signaling [37]. Conversely, CCN3 overexpression in endothelial cells delays IκBα degradation, inhibits p65 nuclear translocation, and reduces VCAM‐1 and ICAM‐1 expressions [16]. These divergent findings likely reflect cell type‐ and stimulus‐specific effects. Given the central role of NF‐κB in TNF‐α and IL‐1 production, CCN3‐mediated regulation of this pathway may be relevant to RA inflammation [84]. However, most evidence comes from non‐RA models, and the role of CCN3‐NF‐κB crosstalk requires validation in RA synovial fibroblasts and immune cells.

#### 4.4.2. Wnt/β‐Catenin Signaling Pathway

The Wnt/β‐catenin signaling pathway plays a pivotal role in cartilage degradation and bone remodeling in arthritis [85–87]. In the context of RA, suppression of this pathway has been shown to exacerbate bone erosion [88]. CCN3 has been reported to inhibit Wnt/β‐catenin signaling in several autoimmune diseases. Specifically, CCN3 suppresses the pathway by downregulating key transcriptional mediators, such as LEF1 and TCF, thereby contributing to a homeostatic feedback mechanism that limits ECM production, as observed in systemic sclerosis [70]. In RA, CCN3 has been implicated in the inhibition of osteoblastogenesis, primarily by suppressing BMP‐induced Smad1/5/8 phosphorylation and reducing β‐catenin expression and transcriptional activity [69]. Overexpression of CCN3 inhibits classical osteoblast markers, including alkaline phosphatase activity and osteocalcin mRNA expression. Mechanistically, CCN3 directly interacts with BMP‐2, which may underlie its inhibitory effect on BMP signaling. This interaction has been validated using GST pulldown assays, which demonstrated specific binding of CCN3 to BMP‐2 but not to Wnt3a or its coreceptor LRP6. In vivo, transgenic mice expressing *Nov* under the control of the human osteocalcin promoter exhibited decreased bone mass, particularly marked reductions in trabecular bone volume, consistent with impaired osteoblast function. These findings suggest that CCN3 may indirectly affect Wnt/β‐catenin signaling by antagonizing BMP‐2 activity, thereby inhibiting osteoblast differentiation and contributing to bone loss.

#### 4.4.3. Other Relevant Pathways

The PI3K/Akt pathway is closely associated with cell survival and the maintenance of inflammation in RA [89–91]. In SW1353 cells, treatment with recombinant CCN3 significantly attenuated IL‐1β‐induced phosphorylation of PI3K, Akt, and mTOR, indicating a direct inhibitory effect of CCN3 on the PI3K/Akt/mTOR pathway activation [52]. In contrast, Chen et al. [71] reported that CCN3 stimulation enhanced the phosphorylation of FAK and Akt, thereby suppressing miR‐608 and promoting the expression of osteogenic transcription factors Runx2 and Osterix.

Multiple key cytokines in RA, including IL‐6 and IFN‐γ, exert their biological effects via the JAK/STAT signaling pathway. The clinical efficacy of selective JAK inhibitors, such as tofacitinib, has confirmed the pathological relevance of this pathway in RA [92]. Although direct modulation of JAK/STAT signaling by CCN3 has not been conclusively demonstrated, clinical data indicate a significant positive correlation between CCN3 and IL‐6 levels (*p* < 0.05) [31], suggesting a potential functional link. Mechanistically, IL‐6 activates JAK kinases through receptor complex formation, leading to the phosphorylation of STAT1/STAT3 heterodimers at the Y705 site. The gp130 subunit acts as a central signaling scaffold, recruiting JAKs via its Box3 domain and triggering STAT3 homodimerization, nuclear translocation, and transcriptional activation of downstream genes such as RANKL and MMP‐9, which are involved in synovial fibroblast hyperproliferation and osteoclast differentiation [72].

CCN3 has also been implicated in the regulation of the TGF‐β/BMP signaling axis. TGF‐β plays dual roles in RA, mediating both proinflammatory and tissue‐reparative responses. CCN3 has been shown to antagonize BMP ligands, such as BMP‐2, thereby modulating Smad‐dependent signaling [69]. Recombinant CCN3 enhances TGF‐β‐responsive gene expression in macrophages and synovial cells, and TGF‐β signaling promotes cellular senescence via cyclin‐dependent kinase inhibitors, driving SASP development to sustain chronic inflammation in RA [47, 73, 74]. Furthermore, TGF‐β has been implicated in alternative pathways of osteoclastogenesis, promoting bone damage in RA [75].

The role of Notch signaling in RA includes the regulation of synovial angiogenesis and the differentiation of bone marrow‐derived cells. CCN3’s interaction with the Notch pathway remains controversial. Some studies suggest that CCN3 binds to Notch1 via its C‐terminal domain, positively regulating Notch activity and suppressing myogenic differentiation [76]. CCN3 overexpression has been reported to enhance levels of cleaved Notch1, the active intracellular form, thereby increasing the abundance of the Notch intracellular domain (NICD) [54], which is consistent with the findings reported by Minamizato et al. [77] However, in osteoblastic cell lines (ST‐2 and C2C12), CCN3 overexpression failed to activate Notch signaling. Instead, it inhibited the transcriptional activity of a 12xCSL‐Luc reporter construct containing CSL‐binding sites and reduced the expression of the canonical Notch target gene HES1. These results suggest that CCN3 may also interact with the extracellular domain of Notch receptors, potentially influencing neovascularization or bone remodeling in RA [69, 78].

The MAPK pathway, including p38 MAPK, extracellular signal‐regulated kinases (ERK1/2), and c‐Jun N‐terminal kinase (JNK), represents another critical signaling network implicated in RA pathogenesis [93, 94]. In vitro stimulation of osteoblasts with CCN3 significantly reduced the phosphorylation of ERK1/2 and p38 MAPK [79]. Su et al. [78] further demonstrated that CCN3 inhibits BMP9 expression and downstream BMP/Smad and BMP/MAPK signaling, thereby suppressing osteogenic differentiation. Mechanistically, this effect is mediated in part by reciprocal inhibition between CCN3 and DLL1, the canonical Notch ligand, resulting in reduced transcription of shared target genes such as Hey1.

In summary, CCN3 does not appear to act solely through a single canonical pathway. Rather, it functions as a signaling integrator, interacting with multiple receptors and ligands to modulate a range of pathways—including PI3K/Akt, JAK/STAT, TGF‐β/BMP, Notch, and MAPK—that are central to inflammation, senescence, angiogenesis, and bone remodeling in RA.

## 5. Discussion

Recent studies suggest that CCN3 plays a complex role in joint diseases, with the potential for a “double‐edged sword” effect. On the one hand, in OA models, CCN3 exhibits beneficial effects by protecting cartilage and counteracting degeneration. On the other hand, in inflammatory arthritis such as RA, the level of CCN3 is positively correlated with inflammation and disease activity, suggesting its involvement in the proinflammatory damage process of the disease. This phenomenon raises a controversy: does CCN3 promote inflammatory damage, or does it protect joint tissue to some extent? One possible explanation is that the effect of CCN3 is context‐dependent: in joint diseases primarily driven by mechanical stress, such as OA, CCN3 tends to protect tissues, whereas in immune‐mediated RA, the pathogenesis involves a vast and complex network of immune cells, cytokines, chemokines, proteases, and MMPs. High levels of CCN3 may reflect the body’s attempt to control inflammation, but this is accompanied by tissue damage, representing a complex balance. Currently, no definitive conclusion can be drawn, and further functional experiments and animal models are needed to decipher the net effect of CCN3. In contrast to these findings, CCN3 has been observed to limit osteoblastic differentiation and accelerate cartilage aging in OA, while in RA, it is noted to negatively regulate certain inflammatory factors. This may suggest that CCN3 is both driven by inflammation and feedback‐regulated by it, with distinguishing the primary and secondary causal factors being a current challenge. In addition to considering that such differences may stem from diverse experimental methods, antibody sensitivity, or detection objects (mRNA vs. protein), it is important to consider that the specific function of CCN3 depends on the tissue environment and associated signaling pathways. In the complex disease environment of RA, whether CCN3 interacts with other specific factors or feedback mechanisms remains a key question, potentially working together in a cooperative yet opposing manner to influence RA pathogenesis. Some researchers suggest that the balance between CCN family proteins should be considered [95], such as the fibrotic‐promoting CCN2 (CTGF) and the antifibrotic CCN3. The ratio between these two proteins is a crucial factor in diseases like scleroderma. Similarly, in RA, balancing the effects of CCN3 with other CCN family members may be more critical than solely targeting CCN3. Furthermore, the stage of the disease and the environment are also important factors. CCN3 may play different roles in the early and late stages of RA, or its function may differ between acute inflammation and chronic fibrosis. The mechanism of CCN3’s action may also vary across different animal models (e.g., collagen‐induced arthritis vs. gene knockout models), requiring systematic comparative analyses. In conclusion, the mechanism of CCN3 in RA is not yet fully unified, and differences between studies need to be explained through standardized experimental systems and more comprehensive observations.

Future research directions may include: (1) dynamic monitoring of CCN3 expression in different stages of RA: future studies should longitudinally monitor CCN3 expression in clinical samples and animal models, clarifying its dynamic patterns during the early, progressive, and remission phases of RA. For example, the levels of CCN3 in synovial biopsies from early, untreated RA patients could be compared with those in specimens from late‐stage joint replacement, providing insights into the trajectory of CCN3 during disease progression. (2) In‐depth mechanistic research: addressing current unresolved mechanistic issues, more detailed experiments should be conducted. For example, manipulating CCN3 expression (overexpression or knockout) in RA primary synovial cells or animal joints could reveal its impact on the release of inflammatory factors, FLS invasiveness, osteoclastogenesis, and cartilage destruction. This would directly answer whether CCN3 drives or alleviates pathological changes in RA. Further molecular studies should also explore the specific receptors and pathways through which CCN3 exerts its effects, such as identifying the integrin receptors it primarily binds to in the synovium and the downstream signaling cascades involved. (3) Interactions with Other Key Factors: CCN3 does not act in isolation; future research should investigate its interactions with other pathogenic factors in RA. For instance, does CCN3 influence the signaling pathways of classic cytokines such as TNF‐α and IL‐6, or is it associated with the expression of matrix‐degrading enzymes (e.g., MMP13 and ADAMTS5) or bone‐resorbing mediators (e.g., RANKL)? Furthermore, the balance between CCN3 and other CCN family members in the RA microenvironment should be considered to determine if there is functional antagonism or synergy. (4) Evaluation as a Biomarker and therapeutic target: The value of serum or synovial CCN3 levels in disease diagnosis, classification, activity assessment, and prognosis (e.g., radiographic progression) should be evaluated in larger patient cohorts. If CCN3 proves to be a stable and reliable indicator of disease activity, it could be incorporated into RA disease monitoring protocols. From a therapeutic perspective, exploring CCN3‐targeted strategies could be valuable. For example, using CCN3‐neutralizing antibodies, antisense oligonucleotides, or administering recombinant CCN3 protein in animal arthritis models to assess their impact on joint inflammation and destruction. Such studies would provide crucial evidence on whether and how to target CCN3 therapeutically.

## 6. Conclusion

Current studies have highlighted that CCN3 is a molecule worth focusing on in the context of RA. As an important member of the CCN family, CCN3 is upregulated in RA pathogenesis and correlates with disease activity and inflammation. It modulates immune‐inflammatory responses and joint destruction via inflammatory signaling, matrix degradation, and cellular processes. Evidence indicates context‐dependent roles: exacerbating damage or promoting protection/repair, necessitating cautious interpretation of its exact functions in RA. In conclusion, as a key node protein connecting inflammation and tissue remodeling, CCN3 is progressively gaining attention in the basic research on RA. Future interdisciplinary studies are expected to comprehensively reveal CCN3’s role and positioning in RA pathogenesis. Elucidation of its mode of action will enable a more rational evaluation of clinical translation potential. Whether as a biomarker for disease activity or as a novel therapeutic target, CCN3 has the potential to offer new approaches in the diagnosis and treatment of RA. Balancing its proinflammatory and protective effects will be essential in finding the optimal intervention window and strategy, ultimately benefiting RA patients.

## Author Contributions

**Ye Yuan**: writing – original draft, investigation. **Hui Yin**: investigation. **Mengya Jiao and Lihua Duan**: writing – review and editing, conceptualization.

## Funding

This work was supported by the National Natural Science Foundation of China (Grants 82371773 and 62363028), the Jiangxi Provincial Health Technology Key Project (Grant 2024ZD003), the Key R&D Program of Jiangxi Province, China (Grant 20232BBG70026), the Jiangxi Provincial Natural Science Foundation (Grant 20252BAC200113), and the Jiangxi Province Key Laboratory of Immunology and Inflammation (Grant 2024SSY06251).

## Disclosure

All authors have read and approved the final manuscript.

## Conflicts of Interest

The authors declare no conflicts of interest.

## Data Availability

Data sharing is not applicable to this article, as no datasets were generated or analyzed during the current study.
